# Switchable CAR T cell strategy against osteosarcoma

**DOI:** 10.1007/s00262-023-03437-z

**Published:** 2023-04-16

**Authors:** Laura Hidalgo, Beatriz Somovilla-Crespo, Patricia Garcia-Rodriguez, Alvaro Morales-Molina, Miguel Angel Rodriguez-Milla, Javier Garcia-Castro

**Affiliations:** 1 Cellular Biotechnology Unit, Instituto de Investigación de Enfermedades Raras, Instituto de Salud Carlos III (ISCIII), 28220 Madrid, Spain; 2 Universidad Nacional de Educación a Distancia (UNED), 28015 Madrid, Spain

**Keywords:** Immunotherapy, CAR T, Osteosarcoma, B7-H3

## Abstract

Immunotherapy with chimeric antigen receptor T (CAR T) cells has changed the treatment of hematological malignances, but they are still a challenge for solid tumors, including pediatric sarcomas. Here, we report a switchable CAR T cell strategy based on anti-FITC CAR T cells and a switch molecule conjugated with FITC for targeting osteosarcoma (OS) tumors. As a potential target, we analyzed the expression of B7-H3, an immune checkpoint inhibitor, in OS cell lines. In addition, we evaluate the capacity of an anti-B7-H3 monoclonal antibody conjugated with FITC (anti-B7-H3-FITC mAb) to control the antitumor activity of anti-FITC CAR T cells. The effector functions of anti-FITC CAR T cells against OS, measured in vitro by tumor cell killing activity and cytokine production, are dependent on the presence of the anti-B7-H3-FITC mAb switch. Moreover, OS cells stimulate anti-FITC CAR T cells migration. In vivo, anti-B7-H3 mAb penetrates in the tumor and binds 143B OS tumor cells. Furthermore, anti-FITC CAR T cells reach tumor region and exert antitumor effect in an OS NSG mouse model only in the presence of the switch molecule. We demonstrate that anti-B7-H3-FITC mAb redirects the cytotoxic activity of anti-FITC CAR T cells against OS tumors suggesting that switchable CAR T cell platforms might be a plausible strategy against OS.

**Supplementary Information:**

The online version contains supplementary material available at 10.1007/s00262-023-03437-z.

## Introduction

Sarcomas are a large group of heterogeneous mesenchymal tumors that represent around 10–15% of all pediatric malignancies. Among sarcomas, osteosarcoma (OS) is the most common primary malignancy of the bone with high capacity of local invasion and metastasis that mostly affect children and young adults, peaking at 10–14 years old. Second peak of incidence arise from > 65 years old [1]. The current therapeutic management of OS combines surgical resection and chemotherapy. 5-year overall survival is up to 70% of patients presenting localized disease; however, patients with metastatic, relapse or refractory tumors have a poor prognosis [2]. There have barely been advances in survival and treatment of OS in the last 30 years [3] being urgent the development of new strategies such as immune-based therapies.

Immunotherapies include chimeric antigen receptor-modified T cells (CAR T cells) that recognize and kill tumor cells. CAR T cell therapy has remarkable antitumor effect targeting CD19 or BCMA on hematological malignancies; however, the efficacy against solid tumors is limited. Solid tumors exhibit several challenges for CAR T cells such as (i) the lack of specific tumor antigens to target [4, 5], (ii) difficulties for CAR T cell trafficking and infiltration into the tumor [6] and (iii) a hostile tumor microenvironment that inhibits the function of CAR T cells and leads them to exhaustion [7].

In order to find tumor antigen candidates for treating OS with CAR T cell therapy, several targets have been explored. Among them, HER2, GD2, IL-11Rα, CD276 (B7-H3) and NKG2D ligands (NKG2DLs) have been tested in preclinical studies [8–12]. Folate receptor (FR) has also been proposed through an anti-FITC CAR T cell strategy [13]. Switchable CAR T cell strategies, as anti-FITC and other anti-tag CAR T cells, are modular approaches where CAR T cell activity is controlled by intermediary switch molecules [14]. These switches or adaptors target tumor-associated antigens and are linked with an epitope that selectively binds the CAR, mediating the interaction between CAR T cells and tumor cells [15–17]. These strategies aim targeting different tumor antigens and improving the safety of conventional CAR T cell therapy. Here, we propose to use an anti-B7-H3 monoclonal antibody conjugated with FITC (anti-B7-H3-FITC mAb) as an adaptor to target B7-H3^+^ OS tumor cells using anti-FITC CAR T cells.

B7-H3 is a very promising target for immunotherapies for solid tumors because it is highly expressed on different tumor types [11]. However, B7-H3 expression is limited on normal tissues. Member of B7 family, B7-H3 acts as an immune checkpoint protein inhibiting the function of T cells [18, 19]. It is overexpressed in OS patients, and it has been associated with tumor aggressiveness and metastasis [20]. There are several preclinical studies addressing the antitumor effect of mAb and antibody–drug conjugates [21, 22] against B7-H3. CAR T cells targeting B7-H3^+^ OS tumor cells have also been studied with interesting results [11]. In addition, a few clinical trials targeting B7-H3 in solid tumors, including OS, are currently running (NCT04483778, NCT02982941, NCT04897321 and NCT04864821).

In this report, we use FITC-labeled anti-B7-H3 mAb as a strategy for controlling CAR T cell activity. We demonstrated that, in a NSG model, anti-B7-H3 mAb reaches the tumor and binds OS cells expressing B7-H3. Additionally, anti-FITC CAR T cells traffic to tumor site and exert their antitumor function in a specific manner, only when anti-B7-H3-FITC mAb is present.

## Material and methods

### Anti-B7-H3 monoclonal antibody production

8H9 hybridoma, secreting anti-B7-H3 mAb, was purchased from ATCC (USA) and cultivated according to the recommendation of the supplier. mAb was purified from the culture supernatants by affinity chromatography column with protein G-Sepharose (BioRad) and dialyzed in PBS. Concentration was determined by Bradford assay. mAb was conjugated with FITC (anti-B7-H3-FITC mAb) or Alexa Fluor 647 using a Fast kit of conjugation (Abcam).

### Cell lines

The human OS cell lines 143B, MNNG/HOS, SAOS-2 and U-2 OS were cultured in DMEM (Lonza) containing 10% fetal bovine serum (FBS), 100 IU/ml of penicillin/streptomycin (Lonza) and glutamine (2 mM). OS cell lines were regularly tested for Mycoplasma detection (Lonza). For authentication, OS cell were examined by STR analysis (IIBM, Madrid).

For in vitro assays, OS cell lines were transduced with lentiviral vectors in order to express GFP, using PL-SIN-EF1α-EGFP plasmid from Addgene (Ref: 21320).

### PBMC and T cell isolation

Leukocyte Reduction System (LRS) cones from healthy donors were provided by the Biobank Hospital Universitario Puerta de Hierro Majadahonda (HUPHM)/Instituto de Investigación Sanitaria Puerta de Hierro-Segovia de Arana (IDIPHISA) (PT17/0015/0020 in the Spanish National Biobanks Network), and they were processed following standard operating procedures with the appropriate approval of the Ethics and Scientific Committees. Human peripheral blood mononuclear cells (PBMCs) were isolated by Lymphoprep (Alere Technologies AS) density-gradient centrifugation and subsequently cryopreserved. For the CAR T cell generation, isolated T cells were obtained by negative selection using a Pan T Cell Isolation Kit (Miltenyi Biotech) from frozen PBMCs.

### Lentiviral vector generation and anti-FITC CAR T cells transduction

Anti-FITC CAR construct was kindly provided by Dr. Michael Jensen from Seattle Children´s Research Institute, Washington, USA. This second-generation fully human CAR construct contains a (i) fully human anti-FITC scFv (clone E2), (ii) an IgG4 hinge-CH2(L235D,N297Q)-CH3 spacer, (iii) a CD28-transmembrane domain, (iv) a 4-1BB/CD3z signaling domain and (v) a cell-surface human EGFRt tag, as described previously [13].

Lentiviral vectors were produced in HEK293T cells co-transfected with anti-FITC CAR single chain variable fragment (scFv) encoding plasmid, the packaging plasmid (PsPAX2) and the envelope plasmid (VSV-G). The supernatants were collected 48 h after transfection, filtrated through a 0.45 µm filter (Millipore) and ultracentrifugated at 23,000 rpm for 2 h at 4 °C.

Purified T cells were activated overnight using Dynabeads Human T-Activator CD3/CD28 (Gibco) at 1 bead: 2 T cells ratio, in X-VIVO 15 media (Lonza) supplemented with 250 U/ml of IL-2 (Miltenyi Biotech). Next day, activated T cells were transduced with lentiviral particles using MOI 2. Spin infection was performed by centrifugation at 2000 g during 2 h at 32 °C. 6 h later, fresh media was added. CAR T cells were maintained at 10^6^ cells/ml in X-VIVO 15 media supplemented with 250 U/ml of IL-2. Media was refreshed every 2 days. In order to expand CAR T cells, beads were removed at day 7. From then, CAR T cells were cultured in X-VIVO 15 media supplemented with 40 U/ml of IL-2. Experiments were performed with resting CAR T cells from day 11 after transduction. For in vitro experiments, resting CAR T cells were cryopreserved and lately thawed and maintained for at least 24 h in X-VIVO 15 media supplemented with 40U/ml of IL-2 prior assays.

### Cell-mediated cytotoxicity in vitro assay

OS cell lines expressing GFP were seeded at 2 × 10^4^ cells per well into a P24-well plate. After 24 h, cells were incubated with PBS or 100 μg/ml of anti-B7-H3-FITC mAb for 30 min. Tumor cells were washed and cocultured with anti-FITC CAR T cells, at different ratios effector: target cells (E:T; 1:4, 1:2, 1:1, 2:1 and 4:1) in X-VIVO 15 supplemented with 40 U/ml of IL-2. After 48 h, cells were collected and stained with anti-CD45 mAb and 7AAD (Biolegend) and analyzed by flow cytometry. The number of live tumor cells (GFP^+^7AAD^−^) preincubated with anti-B7-H3-FITC mAb was normalized to the number of live tumor cells preincubated with PBS. Supernatants from cocultures were collected and IFNγ and TFNα levels were tested by ELISA (Biolegend).

For the experiments combining different mAbs, we followed similar protocol. Briefly, after 24 h of 143B seeding, tumor cells were incubated with anti-B7-H3-FITC mAb, anti-CD29-FITC, anti-CD166-FITC or anti-CD105-FITC alone or anti-B7-H3-FITC mAb in combination with each of the other mAb-FITC. Number of live tumor cells were analyzed by flow cytometry.

### In vitro chemotaxis assay

Chemotaxis of anti-FITC CAR T cells in response to tumor cells was assayed in 5 μm diameter pore size transwell chambers. 10^5^ OS cells per well were seeded in a P24-well plate in DMEM 10% FBS and allowed to adhere for 4 h. Anti-FITC CAR T cells and tumor cells were starved during 16 h in DMEM supplemented with 0.1% bovine serum albumina (BSA) (Sigma-Aldrich). Transwell upper chamber was coated with fibronectin (1 µg/ml) at 4 °C for 16 h. Before assay, OS cells were incubated for 30 min with PBS or 100 µg/ml of anti-B7-H3-FITC mAb. Then, 10^5^ anti-FITC CAR T cells were added to the transwell upper chamber, and migration was allowed to proceed for 3 h in DMEM 0.1% BSA. Migrated cells were analyzed by flow cytometry. Migratory capacity was calculated as ${\text{Input}} = 100 \times \left( {\text{CAR T cells migrated}} \right)/\left( {\text{CAR T cells seeded}} \right)$ and ${\text{Migration index}}\;{ = }\;\left( {\text{migrated CAR T cells to OS cells}} \right){/}\left( {\text{migrated CAR T cells to basal conditions}} \right)$.

### Mice

NOD.Cg-Prkdc^scid^ Il2rg^tm1WjI^/SzJ (NSG) mice (Jackson) at ages ranging from 8 to 12 weeks were used for in vivo experiments. All procedures and animal care were performed at the Instituto de Salud Carlos III (Madrid, Spain). All protocols were approved by the pertinent ethical committees and carried out in accordance with the guidelines of the European directives and Spanish laws (PROEX 133.7/21).

Xenograft models of OS tumors were generated by injecting 10^6^ 143B cells subcutaneously in the right flank of mice. In vivo assays were initiated at day 14 after inoculation.

### In vivo mAb B7-H3 biodistribution

Mice carrying 143B-cells-derived tumors were divided into three homogenous groups, which were inoculated intraperitoneally with PBS (*n* = 1) or with 100 µg (4 mg/kg; *n* = 3), 50 µg (2 mg/kg; *n* = 3) or 10 µg (0.4 mg/kg; *n* = 3) of anti-B7-H3 mAb (clone 8H9) conjugated with Alexa Fluor 647 (D0). Fluorescence was quantified using the IVIS Spectrum system and Living Image Software (PerkinElmer) after 48 h and 72 h post mAb administration. Tumors were removed at 72 h for ex vivo imaging and processed for flow cytometry analysis.

For time course experiment, 100 µg of labeled mAb (*n* = 10) and PBS control (*n* = 5) were assessed. In this case, imaging analysis was performed at 4, 24, 48 and 72 h after mAb administration. Tumors, lungs, kidneys, spleens and livers were collected at 72 h for ex vivo imaging.

### Anti-FITC CAR T cell homing

For in vivo tracking of CAR T cells, mice were divided into a three homogenous groups and were injected intraperitoneally with PBS (group A, *n* = 5, and group B, *n* = 10) or with 100 µg of anti-B7-H3-FITC mAb (clone 8H9, group C, *n* = 10) (D-1). Next day (D0), anti-FITC CAR T cells were labeled with 8.33 mg/mL DiR buffer (DiIC18(7) or 1,1′-dioctadecyltetramethyl indotricarbocyanine Iodide) for 30 min at 37 °C, according to manufacturer´s instruction (Caliper Lifesciences), prior to inoculation intravenously in mice (group B and C). DiR-labeled anti-FITC CAR T cells homing is determined by the IVIS Spectrum system at 3 h, 7 h, 24 h, 48 h and 6 days post-inoculation and quantified using Living Image Software (PerkinElmer). For ex vivo imaging of DiR-labeled anti-FITC CAR T cells, tumors were collected at different time points.

### Xenograft antitumor responses

For in vivo antitumor response experiments, mice bearing the 143B-cells-derived tumors (*n* = 42) were divided into four homogenous groups. Mice were inoculated intraperitoneally with PBS (groups A and C) or with 100 µg of mAb anti-B7-H3 (clone 8H9) conjugated with FITC (groups B and D) (D-1). Mice were also inoculated intravenously with PBS (groups A and B) or with 5.5 × 10^6^ of anti-FITC CAR T cells (groups C and D) (D0). In addition, PBS (groups A and C) or 50 µg of anti-B7-H3-FITC (groups B and D) were administrated intraperitoneally every two–three days.

Tumor length (L), width (W) and height (H) were measured with a Vernier caliper (Mitutoyo) periodically, and tumor volume (mm^3^) was calculated as follows: $V = \left[ {\left( {{\text{axial diameter length, mm}}} \right) \times \left( {{\text{rotational diameter, mm}}} \right) \times \left( {{\text{sagittal diameter, mm}}} \right)} \right]/2$. Tumor growth was calculated dividing tumor volume measure of each day by the tumor volume measure at the CAR T cell inoculation-starting day (D0). Area under the curve (AUC) was determined from tumor growth following Duan et al., instructions [23]. Antitumor activity was calculated relative to mean tumor growth of PBS group (A). Mice were sacrificed at day 19 after inoculation of anti-FITC CAR T cells.

### Flow cytometry

OS cell lines or CAR T cells were stained with fluorochrome-labeled antibodies against human B7-H3 (EPNCIR122 from Abcam; clone 8H9 from hybridoma cell line), EGFR (R&D Systems), CD45 (Biolegend), CD29 (eBioscience), CD166 (AbD Serotec) and CD105 (Immunostep). Data were analyzed by Quant Analyzer 10 (Miltenyi Biotech) and FlowJo software.

### Statistical analyses

GraphPad Prism 9.1.1 software was used for statistical analysis. Statistical tests used are indicated in figure legends. *p* values of less than 0.05 were considered significant.

## Results

### Osteosarcoma cell lines expressing B7-H3

**Fig. 1:**
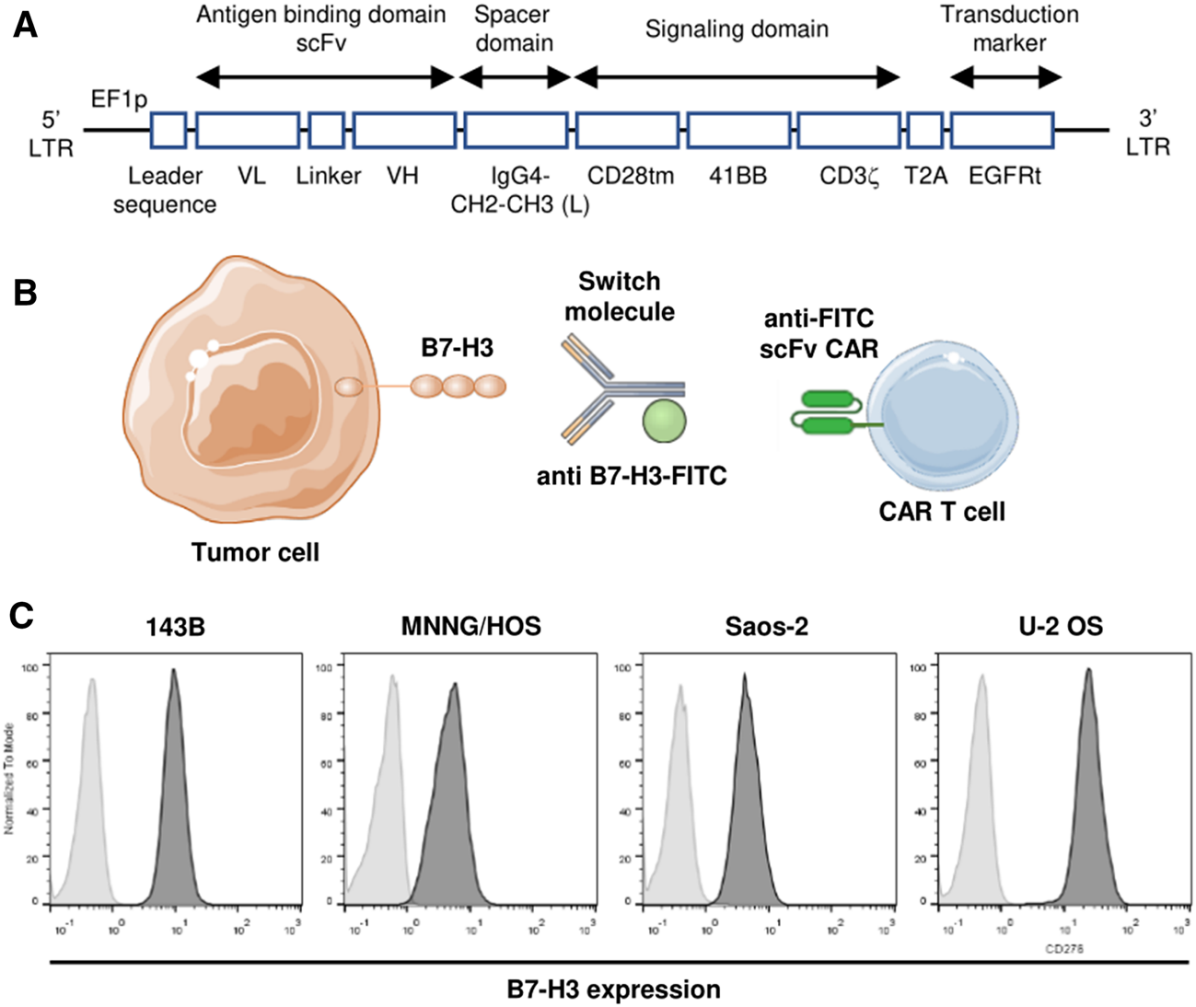
B7-H3 expression on human OS cell lines. **A** Diagram that shows the second-generation fully human anti-FITC CAR construct domains. **B** Schematic representation of our model using a switchable CAR T cell strategy based on an anti-B7-H3 mAb conjugated with FITC and anti-FITC CAR T cells**.** **C** B7-H3 expression is determined on different OS human cell lines by flow cytometry. Soft gray: negative control, dark gray: anti-B7-H3 stained

A second-generation fully human FITC-specific (clone E2) CAR construct (Fig. 1A) was synthesized and provided by The Jensen Laboratory [13].

In order to evaluate whether a mAb against B7-H3 conjugated with FITC (anti-B7-H3-FITC mAb) may be used as a switch for our FITC-specific CAR T system (Fig. 1B), we first studied B7-H3 expression on several OS human cell lines. Analysis by flow cytometry showed that B7-H3 was mainly expressed on all cell lines evaluated (Fig. 1C); Thus, we considered that B7-H3 could a good target for treating OS with CAR T cell therapy.

### In vitro antitumor activity

**Fig. 2:**
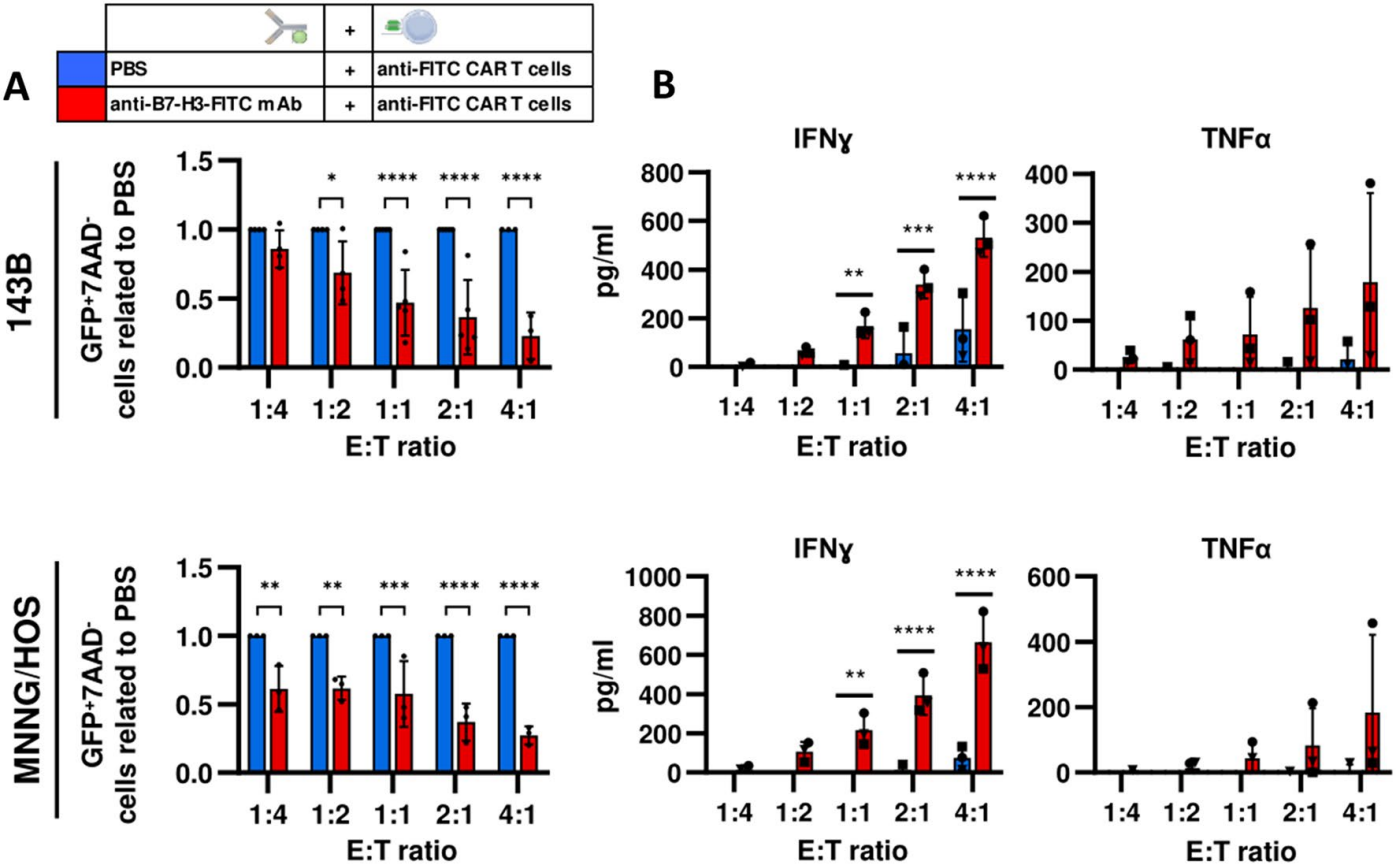
Antitumor activity of anti-FITC CAR T cells. GFP-expressing tumor cells stained (anti-B7-H3-FITC mAb, red) or not (PBS, blue) are cocultured with resting anti-FITC CAR T cells at different effector: target (E:T) ratios for 48 h. **A** Living tumor cells (CD45^−^GFP^+^7AAD^−^) are determined by flow cytometry. The normalization of anti-B7-H3-FITC mAb to PBS is shown. **B** IFNɣ and TFNα levels are tested by ELISA in the cocultured supernatants. All data are represented as mean ± SD of independent experiments with CAR T cells from at least 3 different donors. *, *P* < 0.05; **, *P* < 0.01; ***, *P* < 0.001; ****, *P* < 0.0001 by two-way ANOVA with Sidak post hoc test

We next assessed in vitro the antitumor efficacy of anti-FITC CAR T cells. We first evaluated the number of live OS cells after adding anti-FITC CAR T cells to OS cells preincubated or not with anti-B7-H3-FITC mAb. We quantified the cell-mediated cytotoxicity of anti-FITC CAR T cells by flow cytometry following 48 h of coculture. In presence of the adaptor, anti-FITC CAR T cells showed an enhanced cytolytic response against B7-H3-FITC^+^ OS cell lines (143B and MNNG/HOS cells), even at low CAR T cell: OS cell ratios (Fig. 2A and Fig Suppl 1). The differences in the presence of the mAb were higher as the ratio increased, as shown by calculating the number of live tumor cells related to PBS (Fig. 2A and Fig Supl 1). In addition, we found better anti-FITC CAR T cell activity combining anti-B7-H3-FITC with other mAbs conjugated with FITC targeting CD29, CD166 or CD105 antigens than B7-H3 alone, suggesting that anti-FITC CAR T cells could target different targets at the same time (Fig Suppl 2).

Cytokine release is another important functional characteristic of CAR T cells. We analyzed the concentration of proinflammatory cytokines such as IFNγ and TNFα on supernatants collected from anti-FITC CAR T cells and OS cell lines cocultures. In agreement with a higher cytotoxic capacity, anti-FITC CAR T cells produce significantly greater amounts of IFNγ and TNFα when they are cultured with OS cells that have been previously stained with anti-B7-H3-FITC mAb (Fig. 2C). These results demonstrate that, in vitro, anti-FITC CAR T cells show antitumor activity when OS cells are preincubated with anti-B7-H3-FITC mAb.

### Anti-B7-H3 mAb binds 143B tumor in an in vivo model

**Fig. 3:**
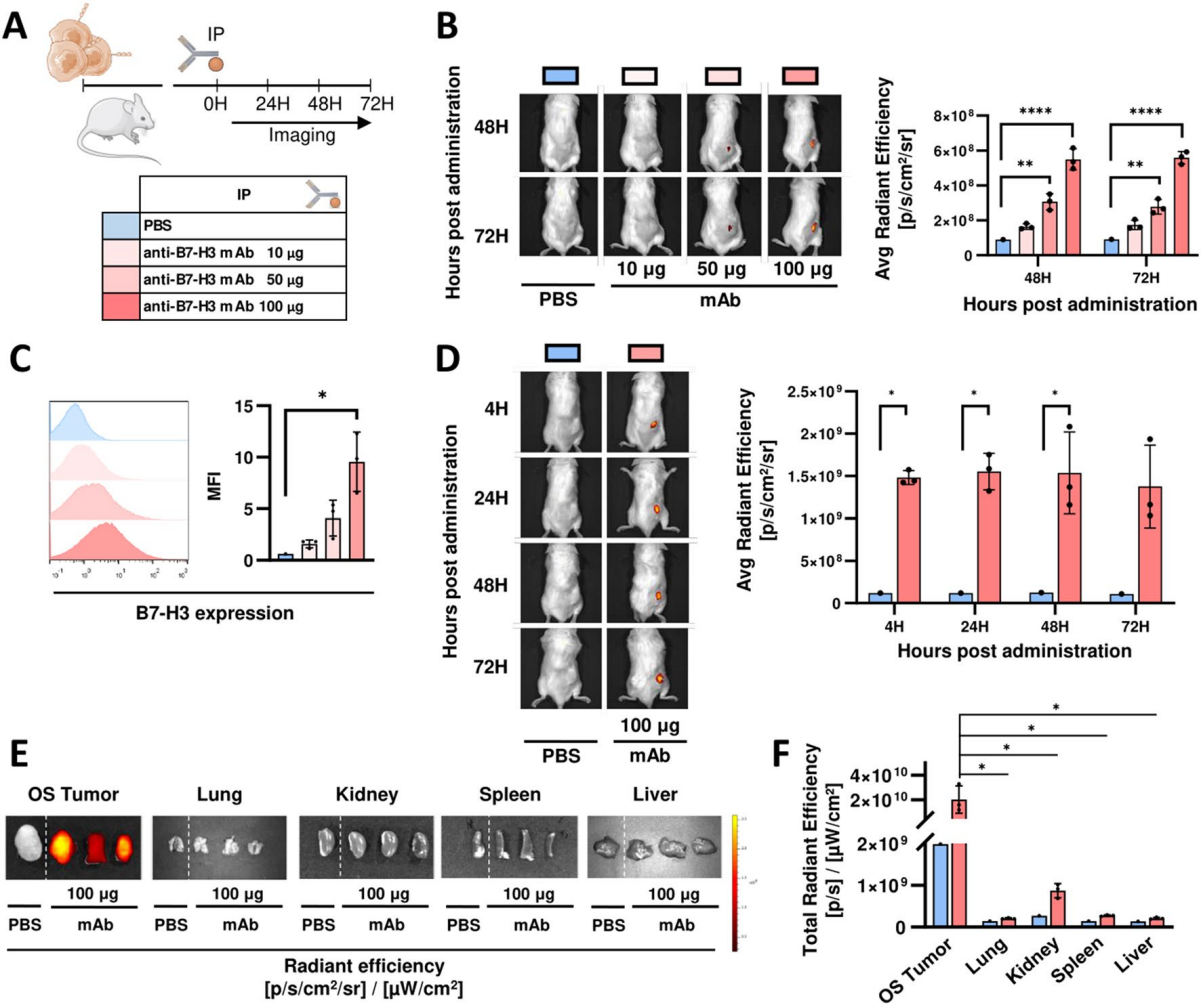
anti-B7-H3 mAb biodistribution in a NSG mice model. **A** Schematic representation of experimental design. PBS or different doses of anti-B7-H3 mAb (clone 8H9) labeled with Alexa Fluor 647 is administrated by intraperitoneal injection (IP) to NSG mice-bearing 143B tumors. **B** and **D** Tumors are imaged at different time points using IVIS imaging system. Representative IVIS image and mean ± SD of average radiant efficiency are shown. *, *P* < 0.05; **, *P* < 0.01; ***, *P* < 0.001 by two-way ANOVA with Tukey (**B**) and Sidak (**D**) post hoc test. **C** B7-H3 expression on ex vivo tumor cells is evaluated by flow cytometry at 72 h. Solid histograms (left) represent the expression of B7-H3 on 143B cells. One representative profile per group is shown. Graph (right) shows the mean fluorescence intensities (MFI) mean ± SD of at least 3 mice. **, *P* < 0.01; by one-way ANOVA with Tukey post hoc test. **E** *Ex* vivo imaging of OS tumors and the organs indicated after 72 h of PBS or anti-B7-H3 Alexa Fluor 647 administration. **F** Quantification of total radiant efficiency of ex vivo imaging (mean ± SD) *, *P* < 0.05 by two-way ANOVA with Sidak post hoc test

Before testing in vivo efficacy of anti-FITC CAR T cells, we studied anti-B7-H3 mAb biodistribution. For imaging analysis in vivo, we first performed fluorescence labeling of anti-B7-H3 mAb with Alexa Fluor 647. Then, we injected intraperitoneally labeled anti-B7-H3-Alexa Fluor 647 mAb at different concentrations into NSG mice implanted subcutaneously with 143B OS tumor cells. We analyze mAb targeting B7-H3 biodistribution using IVIS Lumina II imaging system (Fig. 3A).

Doses of 50 μg and 100 μg are enough for accumulation of labeled mAb in 143B tumor expressing B7-H3 at 48 h. The fluorescent signal is maintained at similar levels at 72 h with both doses (Fig. 3B). Ex vivo analysis of tumors by flow cytometry showed that mAb binds 143B OS cells expressing B7-H3. The percentage and mean fluorescence intensity (MFI) of B7-H3^+^ tumor cells increase with higher doses (Fig. 3C).

In order to analyze whether anti-B7-H3 mAb is accumulated at shorter times, we established a time course of anti-B7-H3-Alexa Fluor 647 mAb biodistribution administrating the highest dose (100 µg). The average radiant efficiencies suggest that mAb targeting B7-H3 reaches the tumor quickly. We could observe a plateau from 4 to 72 h after administration (Fig. 3D). Ex vivo imaging analysis of different organs 72 h post mAb administration shows that tumors treated with mAb display a high fluorescence signal (Fig. 3E and F). These data indicate that anti-B7-H3 mAb accumulated mostly in 143B tumors.

### Anti-FITC CAR T cells show in vitro a high migratory capacity to OS cell lines

**Fig. 4:**
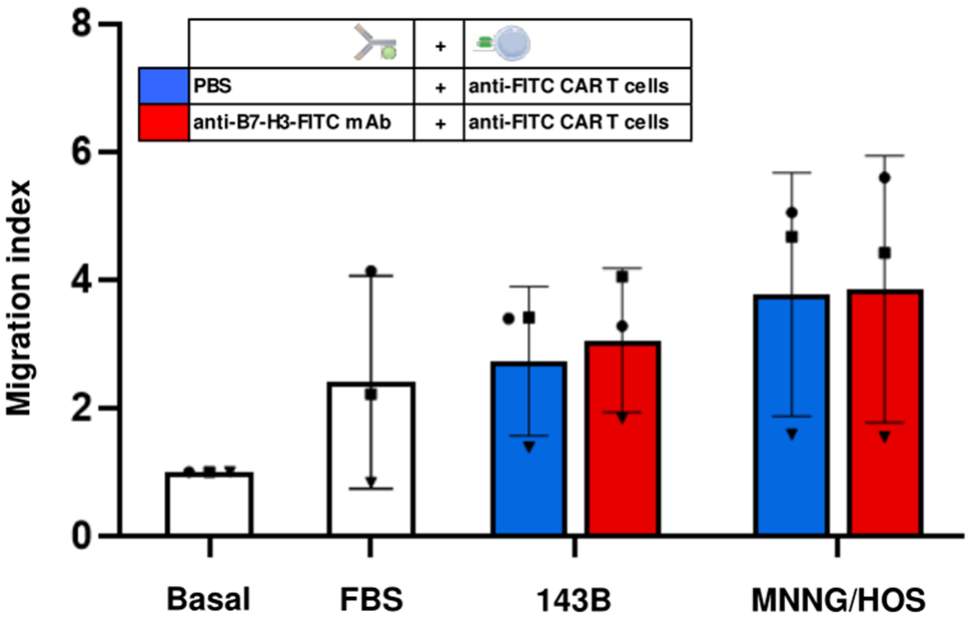
CAR T cell migration in vitro to osteosarcoma cell lines. Chemotaxis assay is performed adding starving CAR T cells in transwell chambers over osteosarcoma cell lines preincubated (red) or not (blue) with anti-B7-H3-FITC mAb. Basal and positive control (FBS) are included. After 4 h, CAR T cells migrated are collected and counted by flow cytometry. Migration index mean ± SD of independent experiments from three different donor is shown

As CAR T cell trafficking to solid tumors is one the main challenges for this therapy, we wanted to evaluate in vitro the migratory capacity of anti-FITC CAR T cells to OS cell lines. For that purpose, we performed chemotaxis assays adding anti-FITC CAR T cells in the transwell chamber over OS cell lines preincubated or not with anti-B7-H3-FITC mAb (Fig. 4).

We measured CAR T cell migration in response to OS cell lines by flow cytometry. Independently of mAb incubation, anti-FITC CAR T cells showed a higher migratory capacity to OS cells comparing to basal condition (Fig. 4). These results suggest that CAR T cell migration is efficient in response to OS cell lines.

### Anti-FITC CAR T cells target 143B tumor-bearing mice

**Fig. 5:**
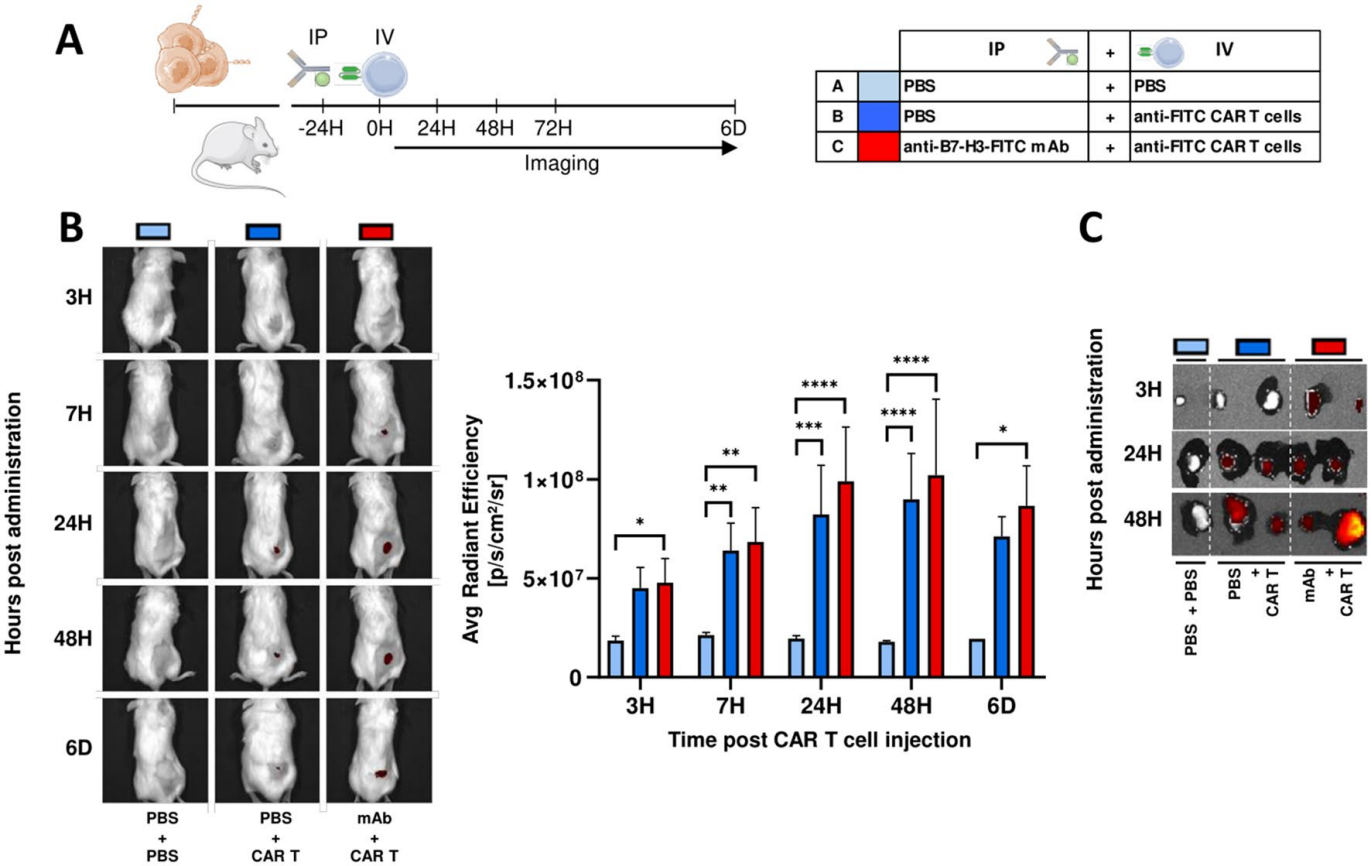
CAR T migration in vivo to 143B tumors. **A** Schematic representation of experimental design. PBS (blue) or 100 μg of anti-B7-H3-FITC mAb (red) is administrated by intraperitoneal injection (IP) (-24H) to 143B tumor-bearing NSG mice. Next day (0H), PBS (light blue = A) or 1 × 10^6^ DiR-labeled CAR T cells (dark blue = B, dark red = C) are intravenously injected (IV). **B** DiR-labeled CAR T cells homing is determinate by IVIS imaging system at indicated time points. Representative images are shown. Average radiant efficiency on tumor region is analyzed, and the mean ± SD of at least three mice is represented. *, *P* < 0.05; **, *P* < 0.01; ***, *P* < 0.001; ****, *P* < 0.0001 by two-way ANOVA with Tukey post hoc test. **C** Ex vivo imaging by IVIS of DiR-labeled CAR T cells on tumors collected at different time points

Once we confirmed the accumulation of anti-B7-H3 mAb at the tumor and the ability in vitro of anti-FITC CAR T cells to migrate to OS tumor cells, we monitored anti-FITC CAR T cell trafficking in 143B tumor-bearing mice. Based on our previous biodistribution results, we first administrated PBS or 100 µg of anti-B7-H3-FITC mAb intraperitoneally. Next day, we injected DiR-labeled anti-FITC CAR T cells intravenously and we tracked the biodistribution at different time points using living imaging (Fig. 5A). We also performed ex vivo imaging of OS tumors harvested at the same points (Fig. 5C). Imaging results indicate that DiR-labeled anti-FITC CAR T cells have already targeted 143B tumors at 3 h, achieving the peak at 24 h (Fig. 5B). We did not observe significant differences in the average radiant efficiency between those mice treated with anti-FITC CAR T cells receiving intraperitoneal injection of PBS or anti-B7-H3-FITC mAb. Ex vivo imaging analysis of 143B tumors (Fig. 5C) confirm the capacity of DiR-labeled anti-FITC CAR T cells to reach the tumor independently of the presence of the switch adaptor.

### Anti-FITC CAR T cells exhibit antitumor activity in an OS mouse model

**Fig. 6:**
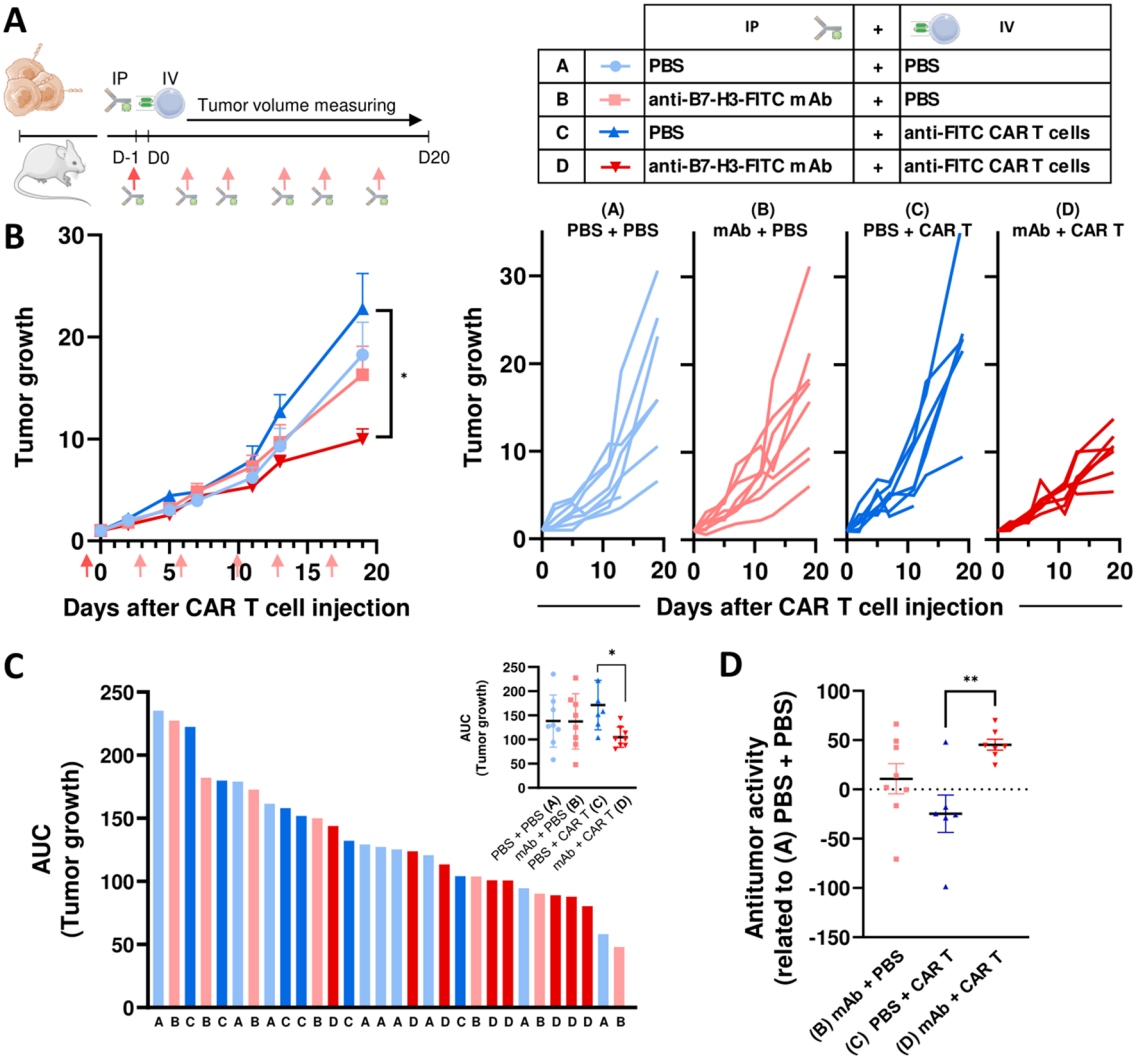
In vivo efficacy of CAR T cell treatment in OS tumors. **A** Schematic illustration of in vivo experimental design. PBS (blue) or 100 μg mAb anti-B7-H3-FITC mAb (red) is administrated by intraperitoneal injection (IP) (D-1) to NSG mice-bearing 143B tumors. Next day (D0), intravenous injection of PBS (light blue = A, light red = B) or 5 × 10^6^ CAR T cells (dark blue = C, dark red = D) is performed (IV). PBS or anti-B7-H3-FITC mAb (50 μg; red arrows) is administrated every 3–4 days. Legend on the right indicates the different conditions. **B** Follow-up of tumor growth in mice treated with different conditions represented as mean + SEM (left) and individual values (right). *, *P* < 0.05 by one-way ANOVA with Tukey post hoc test. **C** Graphs on the left represent the individual areas under the curve (AUC) calculated from tumor growth of mice treated with different conditions. Little graph on the right upper corner represents the AUC expressed as mean + SD. *, *P* < 0.05 by one-way ANOVA with Tukey post hoc test. **D** Graph shows antitumor activity mean + SD of different condition related to PBS + PBS (A) group. **, *P* < 0.01 by one-way ANOVA with Tukey post hoc test

We next assessed the activity of anti-FITC CAR T cells in 143B tumor-bearing mice (Fig. 6A). Measuring tumor size regularly, we observed that the administration of anti-B7-H3-FITC mAb alone does not induce an antitumor effect compared to PBS. Neither the injection of anti-FITC CAR T cells in absence of mAb (Fig. 6B). However, those mice treated with both, anti-B7-H3-FITC mAb and anti-FITC CAR T cells, exhibited a significant delay in the tumor growth (Fig. 6B). In this line, anti-B7-H3-FITC mAb and anti-FITC CAR T cells treatment shows a lower kinetics of tumor growth measured as AUC (Fig. 6C) and a higher antitumor activity (Fig. 6D) compared to the other groups. These results demonstrate that, in our OS model, the antitumor effect of anti-FITC CAR T cells is switch-dependent, only exhibiting antitumor activity when the adaptor is present.

## Discussion

Despite the success of CAR T cell therapy treating hematopoietic malignancies, the results reported on clinical trials for treating solid tumors are no so promising. Some of the key factors limiting the efficacy of CAR T cells on solid tumor are lacking of tumor-specific antigens and immune scape, homing of CAR T cells once infused and an inhibitory tumor microenvironment that lead to CAR T cell exhaustion [7]. Here, we have explored some of these limitations using a universal CAR T cell strategy. As we have mentioned previously, anti-tag CAR T cell activation is controlled by a switch molecule that recognizes tumor-associated antigens [14]. Our results show that using anti-FITC CAR T cells would allow targeting different tumor antigens, simultaneously or sequentially, changing the molecular adaptor. Thus, one of the main advantages of these switchable CAR T cells is avoiding immune evasion and antigen-loss escape preventing relapse or refractory tumors [24]. Antitumor activity of anti-tag CAR T cells has been probed in different types of tumors. Several adaptors conjugated with FITC have been tested including Ab in clinical use [15], Ab-based switches [24] or small molecules such as folate [13]. Actually, a phase I clinical trial with FITC-specific CAR T cells against folate-FITC for osteogenic sarcoma patients is being running (NCT05312411). Here, we propose anti-FITC CAR T cells as a proof of concept of a switchable strategy for targeting B7-H3^+^ OS in preclinical studies. As translation to the clinic of anti-FITC CAR T cells has been disputed because of the immunogenicity of FITC [15], different approaches based on non-immunogenic peptides such as anti-5B9 [16, 25, 26] and anti-peptide neo-epitopes (PNE) [17] CAR T cells have been created. Currently, PNE-based switchable CAR T cell strategy is being evaluated in a phase 1 clinical trial for the treatment of patients with relapsed or refractory B-cell malignancies with promising preliminary results (NCT04450069).

Finding a target highly expressed in the tumor but barely expressed on normal cells is essential for the success of the treatment. In this study, we propose anti-B7-H3 mAb as an adaptor recognizing OS tumor cells. B7-H3 is emerging as a potential target in immunotherapies. Most of pediatric solid tumors, including sarcomas, express B7-H3, and its expression is homogeneous [11, 20]. This fact is very important in CAR T cell therapy since the tumor target heterogeneity is one of the main reasons for therapy failure. In this line, OS is genomically complex and especially heterogeneous, as Sweet-Cordero group has recently confirmed [27].

We demonstrated that B7-H3 might also be a target for universal CAR T cells. All OS cell lines tested in our laboratory express B7-H3 and FITC-specific CAR T cell display antitumor activity in vitro and in vivo only when B7-H3-FITC mAb is present.

Interestingly, B7-H3 has been proposed as an alternative immune checkpoint that leads to T cell exhaustion [28], another of the challenges to overcome for CAR T therapy in solid tumors. The continuous activation, due to persistent antigen stimulation, leads CAR T cells to this stage where they loss antitumor capacity. Immunotherapy against cancer includes the use of immune checkpoint inhibitors. mAb against programmed cell death protein 1 (PD-1) and cytotoxic T-lymphocyte associated antigen 4 (CTLA-4) show clinical success in several tumors. However, PD-1 or CTLA-4 mAb for treating OS has not been reported clinical efficacy [29]. Therefore, it is necessary to find alternatives. mAb and ADC therapy against B7-H3 has been studied in preclinical studies, and Enoblituzumab antibody has been tested in a phase 1 clinical trial in pediatric solid tumor including osteosarcoma (NCT 02982941).

Using an anti-B7-H3 mAb might overcome two obstacles, recognizing tumor cells for CAR T cell activation and enhancing CAR T cell function avoiding inhibition and exhaustion. We hypothesize that combining mAb targeting B7-H3 and universal CAR T cells, might boost CAR T cell function. However, further analysis addressing the function of anti-B7-H3 mAb in combination with universal CAR T cells is necessary. Our results show that treatment with B7-H3 mAb by itself does not have an antitumoral effect in our in vivo model. The fact that NSG mice are immunodeficient model may explain the lack of antitumor effect using B7-H3 mAb in absence of CAR T cells.

Controlling CAR T cell activation is important to limit off-tumor toxicity. Universal CAR T cell activity may be regulated by modifying dose and administration time of the switch. However, our results suggest that a mAb would not be recommended in this sense. Our imaging and flow cytometry results demonstrate that anti-B7-H3 mAb reaches 143B tumors at short times, and the fluorescence signal is maintained at 72 h. Therefore, we consider that CAR T cell toxicity might not be reversed quickly using a mAb strategy, unless FITC-labeled nonspecific mAb or free sodium fluorescein are used [13, 15]. Other molecular adaptors have been proposed with that purpose, for example Ab-based switches with relatively short half-life [30] or PNE switches [17] which are being tested in a phase 1 clinical trial in patients with relapsed/refractory B-cell malignancies (NCT04450069). Preliminary results of this clinical trial show an improvement of the resolution of adverse event holding or reducing the dose of the switch adjusting CAR T cell activity in real time.

Furthermore, CAR T cell homing is critical for the success of the therapy and a major challenge. In this sense, we analyze the ability for anti-FITC CAR T cell administrated intravenously to reach 143B tumors. Through in vivo imaging techniques, we observed that DiR-labeled anti-FITC CAR T cells traffic to tumor site, even when mAb has not been administrated. These results suggest that the presence of the molecular adaptor is not necessary for CAR T cell migration, but it is essential for activation and antitumor function of anti-FITC CAR T cells.

In summary, our studies show that anti-FITC CAR T cells induce antitumor effect in vitro and in vivo and might work as a proof of concept for preclinical studies in solid tumor models. Moreover, we demonstrate that B7-H3 is an interesting candidate for designing adaptors in anti-tag CAR T strategies. Finally, we consider that a switchable CAR T platform might be a versatile tool for OS treatment.

### Supplementary Information

Below is the link to the electronic supplementary material.

Supplementary file1 (DOCX 12 kb)

Supplementary file2 (TIFF 1548 kb)

Supplementary file3 (TIFF 3797 kb)
