# Supplementary material for: Switchable CAR T cell strategy against osteosarcoma
Source: Cancer Immunol Immunother. 2023 Apr 16;72(8):2623–33. doi: 10.1007/s00262-023-03437-z (PMC10361906; doi:10.1007/s00262-023-03437-z)
Supplement: Supplementary file 1 — Supplementary file1 (DOCX 12 kb) [file 262_2023_3437_MOESM1_ESM.docx]

**Fig Suppl 1. Antitumor activity of anti-FITC CAR T cells**. GFP-expressing tumor cells stained (anti- B7-H3-FITC mAb, red) or not (PBS, blue) are cocultured with resting anti-FITC CAR T cells at different effector : target (E:T) ratios for 48 hours. **A)** Living tumor cells (CD3^-^GFP^+^7AAD^-^) are determined by flow cytometry. The normalization of anti-B7-H3-FITC mAb to PBS is shown. All data is represented as mean ± SD of independent experiments with CAR T cells from 2 different donors. *, P < 0.05; **, P < 0.01; ***, P < 0.001; ****, P < 0.0001 by two-way ANOVA with Sidak post hoc test.

**Fig Suppl 2.** **Antitumor activity of anti-FITC CAR T cells combining different mAb-FITC**. 143B GFP-expressing tumor cells are stained with anti-B7-H3-FITC mAb (red), anti-CD29-FITC, anti-CD166-FITC, anti-CD105-FITC alone (yellow) or stained in combination with anti-B7-H3-FITC mAb and other mAb-FITC (orange). Then, tumor cells are cocultured with resting anti-FITC CAR T cells at different effector : target (E:T) ratios for 48 hours. The number of live tumor cells is determined by flow cytometry. All data is represented as mean ± SD of independent experiments with CAR T cells from 2 different donors. *, P < 0.05; **, P < 0.01; ***, P < 0.001; ****, P < 0.0001 by two-way ANOVA with Tukey post hoc test.
