# Supplementary figures and images for: Switchable CAR T cell strategy against osteosarcoma
Source: Cancer Immunol Immunother. 2023 Apr 16;72(8):2623–33. doi: 10.1007/s00262-023-03437-z (PMC10361906; doi:10.1007/s00262-023-03437-z)

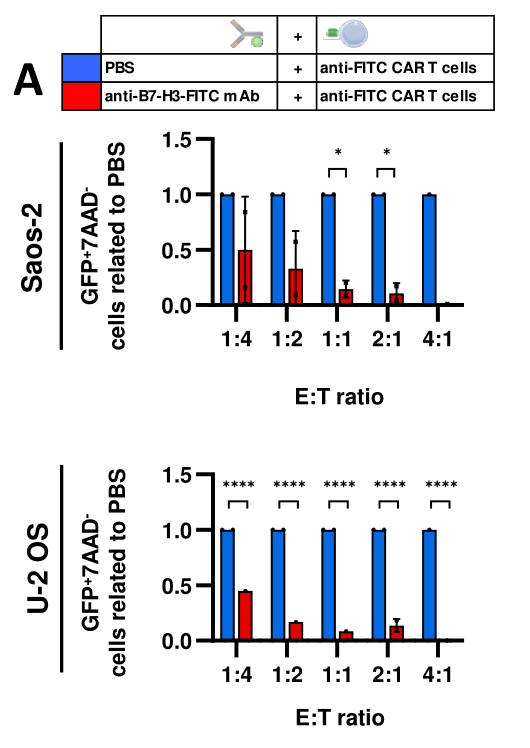

Supplement: Supplementary file 2 — Supplementary file2 (TIFF 1548 kb) [file 262_2023_3437_MOESM2_ESM.tiff]

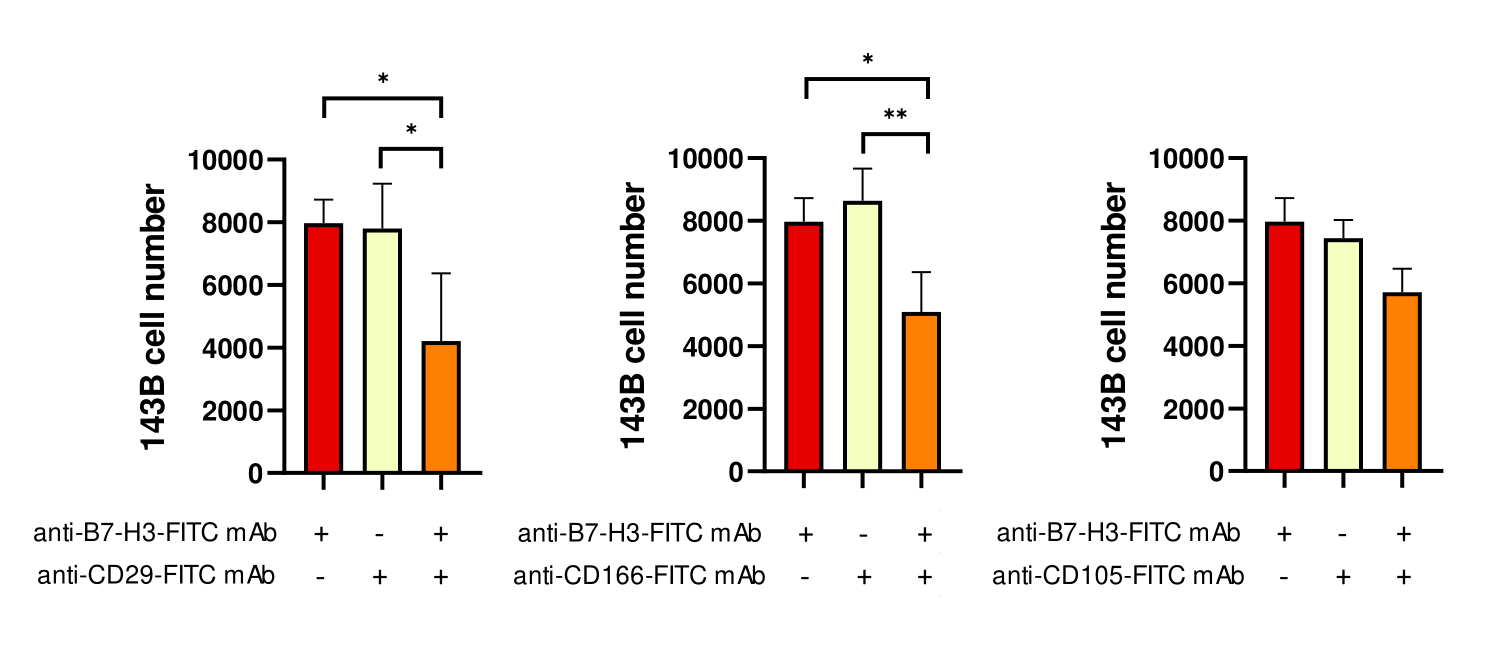

Supplement: Supplementary file 3 — Supplementary file3 (TIFF 3797 kb) [file 262_2023_3437_MOESM3_ESM.tiff]
